# The ecological impact of a bacterial weapon: microbial interactions and the Type VI secretion system

**DOI:** 10.1093/femsre/fuab033

**Published:** 2021-06-22

**Authors:** Ramses Gallegos-Monterrosa, Sarah J Coulthurst

**Affiliations:** School of Life Sciences, University of Dundee, Dow Street, Dundee DD1 5EH, UK; School of Life Sciences, University of Dundee, Dow Street, Dundee DD1 5EH, UK

**Keywords:** Type VI secretion system (T6SS), microbial interactions, inter-bacterial competition, microbiota, host-pathogen interactions, sociomicrobiology

## Abstract

Bacteria inhabit all known ecological niches and establish interactions with organisms from all kingdoms of life. These interactions are mediated by a wide variety of mechanisms and very often involve the secretion of diverse molecules from the bacterial cells. The Type VI secretion system (T6SS) is a bacterial protein secretion system that uses a bacteriophage-like machinery to secrete a diverse array of effectors, usually translocating them directly into neighbouring cells. These effectors display toxic activity in the recipient cell, making the T6SS an effective weapon during inter-bacterial competition and interactions with eukaryotic cells. Over the last two decades, microbiology research has experienced a shift towards using systems-based approaches to study the interactions between diverse organisms and their communities in an ecological context. Here, we focus on this aspect of the T6SS. We consider how our perspective of the T6SS has developed and examine what is currently known about the impact that bacteria deploying the T6SS can have in diverse environments, including niches associated with plants, insects and mammals. We consider how T6SS-mediated interactions can affect host organisms by shaping their microbiota, as well as the diverse interactions that can be established between different microorganisms through the deployment of this versatile secretion system.

## Teaser

Gallegos-Monterrosa and Coulthurst review the distinct functions that the bacterial Type VI secretion system can play during interactions between bacteria and other organisms in diverse ecological niches.

## INTRODUCTION

The microbiota of any ecological niche is formed by the complex community of bacteria, fungi, archaea, protists and viruses that inhabit it. The members of this community interact with one another, establishing relations that can be cooperative, such as producing shared common goods, or competitive, such as fighting for scarce nutritional resources (Adair and Douglas 2017; Bauer et al. 2018; Coyte and Rakoff-Nahoum 2019). Both types of relations are often mediated by the production and secretion of various molecules, from signalling molecules and polymers that mediate cooperation, to toxic proteins and nutrient-sequestering chelators important for effective competition (Little *et al*. 2008; Ghoul and Mitri 2016). The outcome of these interactions determines the ecological fitness of the participants, since it often leads to the death or growth arrest of unfit members of the microbiota (Levine *et al*. 2017; Gore 2018). Among bacteria, this selection pressure has helped to drive the evolution of multiple protein secretion systems, machineries that enable bacterial cells to secrete proteins to the extracellular milieu or translocate them into other cells (Green and Mecsas 2016).

Over the last decade, the Type VI secretion system (T6SS) has emerged as a key player in inter-microbial interactions and a critical determinant of competitive fitness in a variety of contexts. The T6SS is a specialized nanomachine that is widespread among Gram-negative bacteria, where it can be used to deliver proteins directly into adjacent bacteria, eukaryotic host cells, or other microbes, or to release them to the environment (Coulthurst 2019). The T6SS uses a contraction and expulsion mechanism to propel an extracellular puncturing structure with enough force to penetrate the membrane of a neighbouring target cell (Wang *et al*. 2017). The puncturing structure, which resembles a needle, carries a payload of toxic effector proteins that are thus delivered into the target cell, where they can exert their action (Jurėnas and Journet 2020).

**Figure 1.**
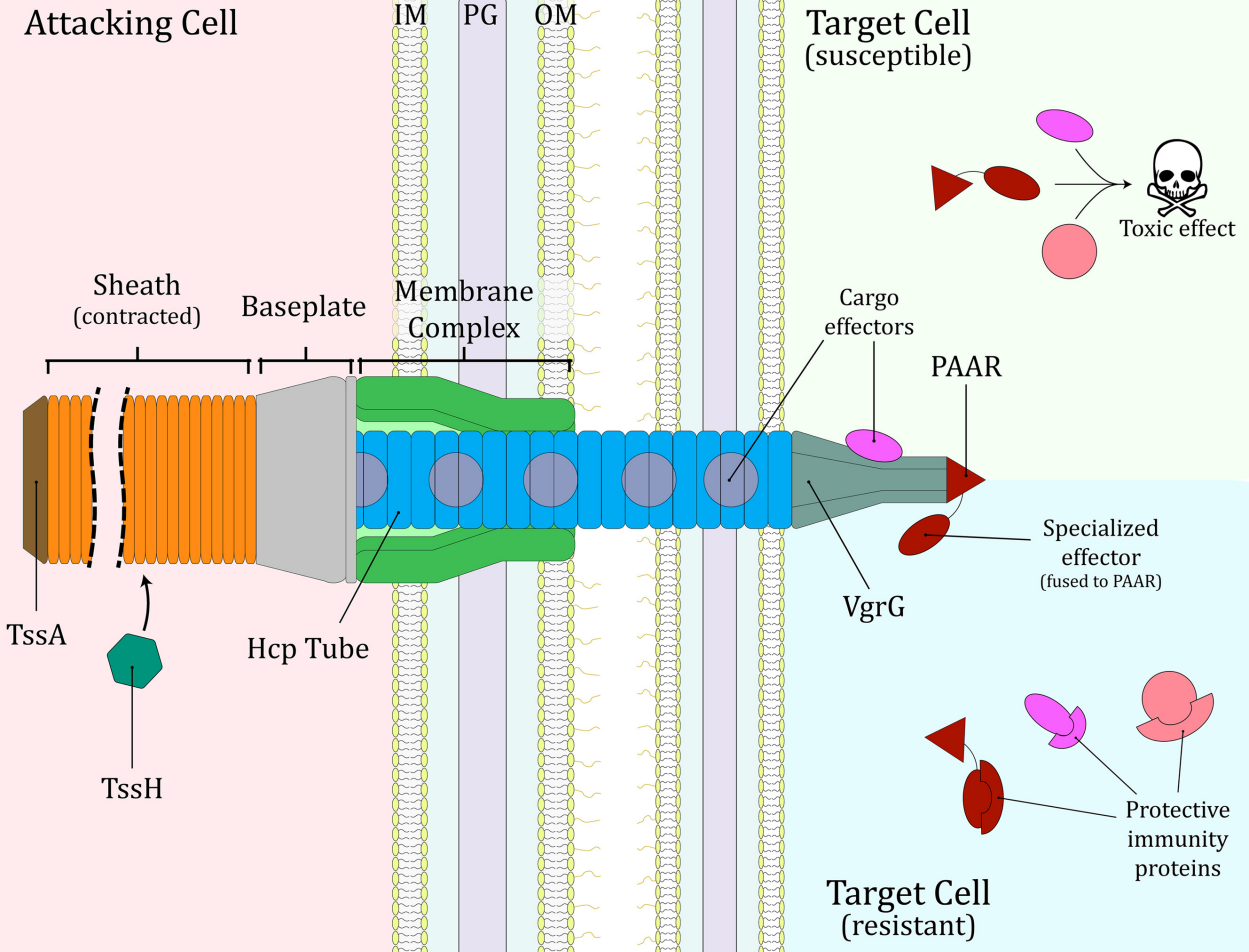
Structure of the Type VI secretion system (T6SS). The system is represented in its contracted state, after a “firing” event. The cytoplasmic baseplate, housing the VgrG-PAAR spike, docks on the membrane complex and acts as a platform for the assembly of the Hcp tube surrounded by the extended contractile sheath. TssA coordinates assembly and extension of the Hcp tube and sheath. During the firing event, contraction of the sheath propels the Hcp-VgrG-PAAR puncturing structure through the membrane complex, out of the attacking cell and into a neighbouring target cell. After contraction, TssH depolymerizes the sheath, facilitating a new round of T6SS assembly and firing. T6SS effectors (cargo and specialized) decorate the puncturing structure and are released into the target cell. Susceptible target cells suffer the noxious actions of the delivered T6SS effectors, while resistant target cells (e.g. sibling cells) possess cognate immunity proteins that bind to the incoming effectors to neutralise them. Note that a Gram-negative bacterium is depicted for the example of a susceptible cell, but the same general mechanism would apply for susceptible eukaryotic cells. IM, inner membrane; PG, peptidoglycan cell wall; OM, outer membrane.

The T6SS is composed of 14 core protein components which form several subassemblies to build the machinery (Fig. 1). The membrane complex (TssJ, L and M) is a bell-shaped structure embedded across the outer and inner membrane of the secreting cell (Durand *et al*. 2015), and it serves to anchor the baseplate of the T6SS (TssE, F, G and K) on its cytoplasmic side. The baseplate houses the tip of the puncturing structure (VgrG and PAAR), and serves as initiation point for the assembly of the contractile sheath (TssB and C) and the main body of the puncturing structure (Hcp) (Cherrak *et al*. 2018; Nazarov *et al*. 2018; Renault *et al*. 2018). The puncturing structure is composed of hexameric rings of Hcp that form a tube-like component, tipped by a VgrG-PAAR ‘spike’, and sits within the rings of the contractile sheath (Pukatzki *et al*. 2007; Shneider *et al*. 2013; Wang *et al*. 2017). TssA coordinates the assembly of the sheath and the tubular Hcp structure by capping the distal end of the extending tube and sheath, although in some systems it may remain attached to the baseplate (Schneider *et al*. 2019; Stietz *et al*. 2020a; Bernal *et al*. 2021). Once the machinery has been assembled, with the sheath in an extended conformation, the T6SS undergoes a “firing” event, namely contraction of the sheath and expulsion of the puncturing structure through the membrane complex and out of the cell (Basler *et al*. 2012). Subsequently, the ATPase TssH (also called ClpV) depolymerizes the contracted sheath, allowing for reutilization of its components to form a new T6SS assembly (Basler and Mekalanos 2012; Kapitein *et al*. 2013). The details of the T6SS firing mechanism have been reviewed recently elsewhere (Cherrak *et al*. 2019; Wang, Brodmann and Basler 2019).

The puncturing structure of the T6SS, composed of Hcp, VgrG and PAAR proteins, serves as a delivery vehicle for a diverse array of effector proteins. These effectors can be classified in two categories based on how they associate with the puncturing structure: “cargo” effectors interact non-covalently with one of the components of the puncturing structure, while “specialized” effectors represent additional homologues of VgrG, Hcp or PAAR proteins with an effector domain covalently fused to the core structural regions (for review, see Jurėnas and Journet (2020)). Effectors secreted by the T6SS show high structural and functional diversity. As examined in detail elsewhere (Hernandez, Gallegos-Monterrosa and Coulthurst 2020; Jurėnas and Journet 2020; Monjarás Feria and Valvano 2020), T6SS effectors have been identified that can function as peptidoglycan hydrolases, phospholipases, DNases, pore-forming proteins, and actin ADP-ribosylases (able to prevent actin polymerisation and induce apoptosis), among many other activities. Importantly, genes that encode antibacterial toxic effectors are accompanied by genes that encode immunity proteins able to bind to their cognate effector, neutralising its action. This protects the toxin-secreting cell from the noxious effects of its own antibacterial effectors and from those that may be delivered by the T6SS of neighbouring sibling cells (Coulthurst 2019; Jurėnas and Journet 2020) (Fig. 1).

Since the formal identification and naming of the T6SS in 2006 (Mougous *et al*. 2006; Pukatzki *et al*. 2006), there has been a remarkable interest in this molecular machine. Many groups have contributed to extensive work to understand the operational aspects of the T6SS mechanism, the regulatory genetic networks that control its expression, and the nature of the effectors that it secretes (Wang, Brodmann and Basler 2019; Jurėnas and Journet 2020). Importantly, there has been a significant change in perspective in terms of the role played by the T6SS: although the initial work that established the foundations of this field suggested that the T6SS was a virulence mechanism directly mediating pathogen-host interactions, further research has shown that the main role of this secretion system is instead to mediate interbacterial conflict and competition. Indeed, in later years an increasing amount of research has focused on investigating the effect of the T6SS on inter-microbial interactions, and how those effects may in turn impact other organisms. In this review, we will examine what is known about the ecological effects of the T6SS on various environments.

## EVOLUTION AND CLASSIFICATION OF THE T6SS

Shortly after the term T6SS was proposed, a bioinformatics analysis of bacterial genomes revealed that gene clusters encoding the core proteins of this secretion system are widespread among Proteobacteria, with more than 25% expected to possess at least one T6SS gene cluster (Bingle, Bailey and Pallen 2008). This study suggested that the presence of multiple T6SS gene clusters in the same genome could be due to horizontal gene transfer (HGT), and proposed a classification system for T6SS gene clusters, dividing them into four groups (named A-D) based on a maximum parsimony analysis of concatenated sequences of TssB and TssC. Shortly afterwards, an examination of 500 bacterial genomes identified 13 T6SS-associated proteins, each forming clusters of orthologous groups of proteins (COGs) that were highly conserved. Comparison of phylogenetic trees of these COGs, now known to represent core components of the T6SS, led to the definition of five groups (I-V) into which T6SS gene clusters could be classified, with these groups matching and expanding the previous classification (Boyer *et al*. 2009).

In 2011, Barret *et al*. analysed 11 T6SS COGs in 34 *Pseudomonas* genomes and proposed a phylogeny where T6SSs are grouped in five clusters (1, 2, 3, 4A and 4B), with a sixth cluster (named 5) containing T6SSs from out-group species used during the phylogenetic analysis. This classification mostly matched the one proposed by Boyer *et al*. (2009), with the main difference being that cluster 4 is subdivided in two groups (Barret *et al*. 2011). A subsequent study expanded the classification by comparing 1127 TssB homologues found in genomes from >300 metagenomic datasets, resulting in a phylogeny with a seventh cluster, ‘FPI’. The FPI group was assigned to a gene cluster found on the *Francisella* pathogenicity island, containing genes sharing similarities with T6SS components but whose architecture is not consistent with a canonical T6SS (Barret, Egan and O'Gara 2013). A further type of T6SS-like gene cluster requiring its own clade in the T6SS classification is found in members of the Bacteroidetes phylum. Using iterative search and protein structural prediction algorithms, Russell *et al*. (2014) identified a cluster of genes in Bacteroidetes genomes that encode putative orthologs of Proteobacterial T6SS proteins. Notably, Bacteroidetes T6SS gene clusters lack homologues of the TssL, TssM, and TssJ proteins, which form the membrane complex in Proteobacteria, and TssA, which orchestrates sheath-tube assembly. However, alternative components TssN, TssO, and TssP may functionally substitute for the membrane complex in Bacteroidetes. Phylogenetic analysis of the T6SS gene clusters of Proteobacteria, *Francisella* and Bacteroidetes showed clear separation of their clusters into three distinct clades, which the authors named T6SS^i^, T6SS^ii^ and T6SS^iii^, respectively (Russell *et al*. 2014).

A consolidated classification for T6SS gene clusters was proposed by Li *et al*. (2015) based on these previous studies. It divides T6SSs into three major classes, summarised in Table 1. Canonical type i T6SS gene clusters, found mostly in Proteobacteria, encode a minimum of 13 T6SS conserved components and are subclassified into six subtypes (i1, i2, i3, i4a, i4b and i5); type ii T6SS gene clusters are carried by the *Francisella* FPI and their genes share limited homology with those of type i; whilst type iii T6SS gene clusters are found in Bacteroidetes and encode components that exhibit distant homology with core components from type i systems. More recently, it was shown that a gene cluster in *Amoebophilus asiaticus* encodes a functional T6SS-like system. The genes of this cluster share low or no homology with those of previously characterised T6SSs (subtypes i, ii and iii), but are similar to those found in a gene cluster of unknown function in the related bacterium *Cardinium hertigii*. Furthermore, the T6SS gene cluster of *A. asiaticus*, like those of Bacteroidetes, lacks homologues of the TssL, TssM and TssJ membrane complex proteins conserved in type i T6SS. Thus, a new T6SS “type iv” was proposed as the correct classification for the cluster found in *A. asiaticus* (Böck *et al*. 2017). An interesting question that emerges from the efforts to classify the T6SS is whether further new architectures or variants of this machinery will be discovered among bacteria that have not yet been studied as intensively as the Proteobacteria, requiring further phylum- or group-specific clades to be assigned.

**Table 1.** Summary of the core components of the major classes of T6SS. Type i T6SSs represent canonical Proteobacterial T6SSs, type ii T6SSs are found in *Francisella*, type iii T6SSs are found in Bacteroidetes, whilst a single type iv T6SS has been reported in *Amoebophilus*. For T6SS components with homologues in bacteriophage T4, the name of the respective phage protein is given in the column ‘T4 phage’. The table summarises information from references throughout the main text, together with Clemens, Lee and Horwitz (2018).

Component	Role	Type i T6SS	Type ii T6SS	Type iii T6SS	Type iv T6SS	T4 phage	Comments
TssA	Promotes sheath polymerisation and co-ordinates sheath and tube assembly	✓					‘Long’ TssA (TsaC) have C-terminal VasJ domain and interact with TagA at the far side of the cell to stabilise the extended sheath. ‘Short’ TssA (TsaB) have a distinct C-terminus and may interact with different accessory proteins to stabilise the sheath from the baseplate.
TssB	Contractile sheath (small subunit)	✓	IglA	✓	✓	gp18	TssB and TssC correspond to the N-and C-terminal domains of gp18 (T4 sheath protein), respectively.
TssC	Contractile sheath (large subunit)	✓	IglB	✓			
Hcp (TssD)	Puncturing structure (tube)	✓	IglC	✓	✓	gp19	
TssE	Baseplate	✓		✓	✓	gp25	
TssF	Baseplate	✓		✓	✓	gp6	
TssG	Baseplate	✓		✓		gp7	
TssH (ClpV)	Sheath disassembly ATPase	✓	ClpB	✓			ClpB (Type ii) is a general chaperone
VgrG (TssI)	Puncturing structure (spike)	✓	VgrG + PdpA	✓	gp27 + gp5	gp27 + gp5	In Type i and iii, VgrG is a fusion of gp27- and gp5-like domains. In Type ii, a short ‘VgrG’ lacks the gp27-like domain which is likely replaced by PdpA.
TssJ	Membrane complex (OM lipoprotein)	✓	IglE				IglE appears to be a functional orthologue of TssJ
TssK	Baseplate	✓	IglD	✓			Similar to siphophage receptor binding protein
TssL	Membrane complex (integral IM protein)	✓	DotU				
TssM	Membrane complex (integral IM protein)	✓	PdpB				
PAAR	Puncturing structure (spike tip)	✓	IglG	✓	✓	gp5.4	
TssN	Membrane complex?			✓			
TssO	Membrane complex?			✓			
TssP	Membrane complex?			✓			
T6SSiv baseplate	Additional baseplate components				✓	gp48, gp6, gp53, gp54	
T6SSiv tail terminator	Control of sheath/ tube length?				✓	gp15	
T6SSiv tape measure	Control of sheath/ tube length?				✓	gp29	
T6SSiv Afp-like	Components of unknown function				✓		Similar to Afp extracellular contraction injection system

Examination of the potential origins of the T6SS and its diversification among bacteria has provided valuable insights to better understand this secretion system. A shared evolutionary origin between major components of the T6SS and the membrane-breaching spike of bacteriophages was first proposed early in the history of the T6SS (Pukatzki *et al*. 2007; Leiman *et al*. 2009; Pell *et al*. 2009). *In silico* and structural analyses of the T6SS have revealed that a number of its proteins show a remarkable similarity to those found in bacteriophages (Table 1). In particular, the proteins that form the puncturing structure of the T6SS (VgrG, PAAR and Hcp) form structures that are virtually identical to those forming the tail spike of bacteriophages T4 and Mu (Leiman *et al*. 2009; Shneider *et al*. 2013). The tail spike is the molecular device used by bacteriophages to break through the bacterial outer membrane during infection (Huang and Xiang 2020). Another T6SS structure with a close resemblance to the phage machinery is the contractile sheath formed by TssBC, which is a tubular assembly highly similar to the contractile tail sheath formed by the gp18 protein of bacteriophage T4 (Lossi *et al*. 2013; Kudryashev *et al*. 2015). Components of the T6SS baseplate have also been identified as structural homologues of T4 bacteriophage proteins, and analysis of function using *in silico* and cryo-electron microscopy methods indicate that assembly of the T6SS baseplate follows a pathway similar to that of the T4 baseplate (Taylor *et al*. 2016; Cherrak *et al*. 2018). It is also important to note the differences between the T6SS and the bacteriophage injection machinery. The most striking one is perhaps the membrane complex, which is exclusive to the T6SS and plays a similar role to a bacterial phage receptor by providing a docking site for the baseplate, in this case orientated towards the cytoplasm instead of the extracellular space (Zoued *et al*. 2016; Nguyen *et al*. 2017; Cherrak *et al*. 2018). Another important difference is the dynamic function of the T6SS, which undergoes cycles of assembly, firing and disassembly (Basler and Mekalanos 2012; Cherrak *et al*. 2019), facilitated in part by the sheath-depolymerising ATPase, TssH, which is exclusive to the T6SS and has co-evolved with its sheath proteins (Förster *et al*. 2014). Finally, bacteriophages use a “tape measure” protein to determine the length of the tail spike (Taylor, van Raaij and Leiman 2018) but T6SSs do not have an equivalent protein. Instead, the length of the contractile sheath and Hcp tube is dictated by cell width (Vettiger *et al*. 2017; Santin *et al*. 2019; Stietz *et al*. 2020b), and their assembly is coordinated by the specialized T6SS protein TssA (Zoued *et al*. 2017; Schneider *et al*. 2019). Thus, the picture that emerges is that certain basic elements of the T6SS machinery (contractile mechanism and puncturing structure) likely share an evolutionary origin with prophages, or even have evolved from one, but other components represent bacterial adaptations that make the T6SS a domesticated, regulated and internally anchored secretion system.

Besides the interest in its origin, recent studies have used *in silico* approaches to examine the diversification of T6SS among bacteria, and to show how new T6SS-based competition strategies may emerge. Using a mathematical modelling approach, Smith *et al*. (2020a) showed that the evolution of a ‘defensive’ T6SS that only fires in response to a previous T6SS attack is evolutionarily disadvantageous, and only sustainable under specific interaction criteria where the retaliating bacterium survives the initial attack and presents a strong toxic response of its own. Furthermore, these authors also showed that the contact-dependent nature of the T6SS can become a hindrance to its own activity by forming barriers of dead cells around the T6SS-active cell, and thus the evolution of effectors that lead to cell lysis is favoured (Smith *et al*. 2020b). As above, examination of T6SS gene clusters among Proteobacteria, Bacteroidetes, and *A. asiaticus* has revealed sufficient diversity for distinct types of T6SS to be clearly established and classified. The fact that the T6SS of Bacteroidetes and *A. asiaticus* lack homologues of the proteins required to establish the membrane complex of the T6SS in Proteobacteria (Russell *et al*. 2014; Böck *et al*. 2017) is particularly interesting, as it suggests multiple and parallel evolutionary origins for the T6SS from bacteriophage elements.

## ECOLOGICAL IMPACT OF THE T6SS IN DIVERSE NICHES

**Figure 2.**
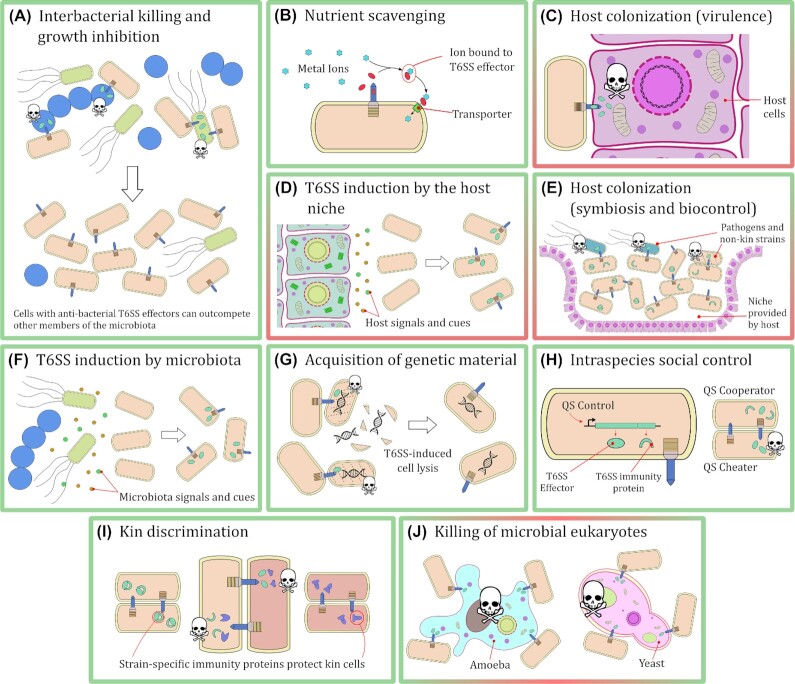
Known types of cellular interactions involving the T6SS. **(A)** The T6SS enables bacteria to outcompete other members of the microbiota by killing them or inhibiting their growth. **(B)** Effectors secreted by the T6SS (red ellipses) can sequester nutrients present in the environment and facilitate their transport into the secreting cell. **(C)** Bacteria can deliver T6SS effectors to host cells as a virulence mechanism during colonization. **(D)** Signalling molecules and metabolites found in the host environment can induce expression of the T6SS in bacteria. **(E)** Bacteria can use the T6SS to outcompete other members of the microbiota and colonize a niche provided by a host. Subsequently they can use the T6SS to prevent invasion by pathogenic organisms. **(F)** Members of the microbiota secrete signalling molecules and metabolites that can induce expression of the T6SS in bacteria. **(G)** Bacteria can use T6SS-delivered effectors to lyse related cells (non-kin); DNA released by this mechanism (black double helix) can then be acquired by the secreting cells. **(H)** Quorum sensing systems can enable policing of cheater cells by positively controlling the expression of T6SS effector and immunity genes (green boxes following black arrow). **(I)** Minor differences in T6SS effectors and immunity proteins allow otherwise isogenic cells to differentiate and kill each other. (**J)** The T6SS allows bacteria to kill microbial eukaryotes that may be competitors and predators. Throughout all panels, green ellipses represent T6SS effectors and green arcs represent T6SS immunity proteins. Panels with a green frame indicate interactions among bacteria only. Panels with red/green frames indicate interactions between bacteria and eukaryotes. Skulls represent cell death or growth inhibition by delivered T6SS effectors.

The variety of T6SS-delivered effectors and their diverse function means that this secretion system plays an important role in shaping the microbiota of many ecological niches. Bacteria that possess a T6SS are commonly able to target bacterial competitors and arrest their growth or even cause cell death, thus changing the population ratios of the microbiota (Coulthurst 2019). Importantly, these competitors can be close genetic relatives, for example strains of *Proteus mirabilis* are able to discriminate and kill each other based on minor differences among otherwise conserved T6SS effectors (Alteri *et al*. 2017). A more subtle mechanism by which the T6SS is used to shape microbial communities is by controlling the availability of key nutrients. Recently, T6SS-dependent effectors have been identified that are capable of sequestering metal ions, to be then acquired by the producing bacterium. *Burkholderia thailandensis*, for example, can produce and secrete TseM, an effector capable of binding to extracellular manganese ions which are then imported back into *B. thailandensis* cells (Si *et al*. 2017b). This function of T6SS effectors can lead to depletion of limited nutrients in a given environment, leading to loss of fitness in those members of the microbiota that are unable to successfully compete for the same ions or secure an alternative nutrient source. Another mechanism by which the T6SS can shape a microbial community is by affecting its eukaryotic members. In many aquatic and soil environments, amoebal predation of bacteria (also known as ‘grazing’) is a common phenomenon (Shi *et al*. 2020). Faced with this threat, some bacteria have evolved T6SS effectors that are able to protect them from amoebal predation (Pukatzki *et al*. 2006; Bayer-Santos *et al*. 2018). Finally, it has been shown that the T6SS can participate in mechanisms of social control within an isogenic bacterial population, expanding the perspective of this system beyond the killing of non-sibling cells (Majerczyk, Schneider and Greenberg 2016). The different kinds of interactions that can be mediated by the T6SS, and that will be discussed as we consider the ecological impacts of this system, are summarised schematically in Fig. 2.

Our general understanding of how the T6SS shapes microbial communities and their ecology continues to expand as new effectors are discovered and characterized, and through the rise of metagenomic studies of microbiotas. In the following sections we discuss the impact that the T6SS can have on diverse ecological niches. Importantly, the capacity of the T6SS to deliver multiple and diverse effectors means that it can be deployed against various targets within the same ecological niche. In turn, this indicates that the T6SS can play a complex and nuanced role in shaping the microbiota and one that requires careful analysis.

### Impact of T6SS on the plant microbiota

Bacteria represent a component of the plant microbiota that shows remarkable species diversity and can be found associated with all plant structures, particularly in the root microbiota. Soils possess the highest diversity of bacterial species and the largest average number of cells per volume of any known ecological environment (Schloss and Handelsman 2006; Roesch *et al*. 2007), and the rhizosphere is considered to be the richest niche within this habitat. The rhizosphere is the area immediately adjacent to plant roots, traditionally considered to comprise up to 1500 µm of root-soil interface based on the presence of plant natural products that exude from the roots and diffuse, enriching the soil and creating an optimal environment for the development of a complex microbial community (Hirsch 2004). Although most of the bacterial species associated with plants appear to be commensals, those that are pathogenic can cause significant damage to plants and lead to important economic losses and threats to food security (Savary *et al*. 2012). Several well-known and important plant pathogens possess a T6SS, such as *Agrobacterium tumefaciens* (Wu *et al*. 2008) and *Pseudomonas syringae* (Haapalainen *et al*. 2012). Importantly, in several of these organisms the secretion system does not appear to be primarily used against the plant host, but rather against other members of the microbiota.

The gene cluster that encodes what is known today as the T6SS was first reported in the plant-associated bacterium *R. leguminosarum* in 2003 (known as the *imp* cluster) (Bladergroen, Badelt and Spaink 2003). This study indicated that the T6SS has a inhibitory effect on root nodulation in peas, but it did not establish if the system was responsible for direct interactions between bacteria and plant cells. Studies in plant-pathogenic bacteria have further suggested that this secretion system might be used as a classical virulence factor against plant hosts. Members of the so-called pectolytic Erwinias are responsible for causing soft rot disease in a variety of crop plants (Thomson 1981). In *Pectobacterium (Pc) atrosepticum* 1043, mutations in ECA3438 (*tssK*) and ECA3444 (*tssC*) were shown to significantly reduce virulence compared to the wild-type strain using potato plant infection models where the bacteria are directly inoculated into plant tissues (Liu *et al*. 2008), while expression of its T6SS was shown to be upregulated by potato tuber extracts (Mattinen *et al*. 2008). Additionally, in *Pc wasabiae* SCC3193, deletion of both of its T6SS clusters caused a reduced level of tissue maceration in a potato tuber slice assay where bacteria are inoculated after the potato slices have been sterilized (Nykyri *et al*. 2012). These cases suggest a direct involvement of the T6SS in the plant-pathogen interaction; however, it is important to note that the precise mechanism of how the T6SS contributes to virulence remains to be clarified and the T6SSs effectors responsible have yet to be identified.

Bacteria possessing T6SSs can, in some cases, use this secretion system against both prokaryotic and eukaryotic targets. For example, *V. cholerae* strain V52 has been shown to be able to use its T6SS to outcompete bacteria, avoid amoebal predation, and as an anti-host mechanism by delivering diverse T6SS effectors (Pukatzki *et al*. 2006; Ma and Mekalanos 2010; Unterweger *et al*. 2014). There are several studies indicating that T6SS with anti-bacterial and anti-eukaryotic activity might also be found in some plant pathogens. An initial study of the plant pathogen *A. tumefaciens* C58 reported that a *hcp* mutation leads to a reduction in tumour-formation in the potato tuber slice model (Wu *et al*. 2008). This may suggest a direct role for the T6SS against the plant, although no effectors responsible for this effect have been identified. On the other hand, a subsequent study showed that *A. tumefaciens* C58 produces a family of T6SS DNase effectors that can be used against bacterial competitors such as *P. aeruginosa* PAK in plant models (Ma *et al*. 2014). Importantly, this latter study also highlighted the relevance of the environment in which the bacteria live and compete to the outcome of those interactions: while *A. tumefaciens* was able to outcompete *P. aeruginosa* in an *in planta* model, the opposite was true when the competition took place *in vitro* (Ma *et al*. 2014). This case emphasizes the importance that environmental components and signals from other organisms in the ecological niche have for the shaping of the entire community. Another example of a T6SS that might be able to deliver effectors against bacterial competitors and plant cells is found in *P. aeruginosa*. This opportunistic pathogen possesses three T6SS gene clusters (known as H1, H2 and H3-T6SS) involved in interactions with different organisms, and the expression of these clusters is complex, with both global and cluster-specific regulators responsible for fine-tuning it (Mougous *et al*. 2006, 2007; Allsopp *et al*. 2017). In *P. aeruginosa* PA14 it was shown that deletion of the H2-T6SS or H3-T6SS leads to a decrease of disease symptoms in an *Arabidopsis thaliana* leaf infection model, and that the proliferation of bacterial cells was two orders of magnitude lower compared with wild-type controls (Lesic *et al*. 2009). However again, demonstration of a direct effect, via identification of effectors targeting plant cells, remains to be reported. Many other studies have demonstrated that *P. aeruginosa* strains PAO1 and PA14 can use their T6SSs to deliver effectors against bacterial competitors, including *A. tumefaciens*, *Yersinia pseudotuberculosis, B. thailandensis* and other *P. aeruginosa* strains (Hood *et al*. 2010; Ma *et al*. 2014; Ahmad *et al*. 2019; Wang *et al*. 2020).

Bacteria in the plant microbiota commonly use their T6SS to compete against other bacteria, with varied consequences for the plant host. *In silico* analysis previously showed that various pathogenic subspecies of *Pseudomonas syringae* such as *P. syringae* T1, *P. syringae* pv. *tabaci* ATCC 11528 and *P. syringae* pv. *oryzae* possess two T6SS gene clusters, named HSI-I and HSI-II (Sarris *et al*. 2010). *P. syringae* pv. *tomato* DC3000 is a well-known causative agent of tomato speck disease that depends on its Type III secretion system to disrupt normal plant cell metabolism (Xin and He 2013). Recently, it was shown that the T6SS encoded by the HSI-II gene cluster enables *P. syringae* pv. DC3000 to outcompete several plant-associated bacterial species such as *Dickeya dadantii*, *Pseudomonas savastanoi* and *Xanthomonas euvescatoria* (Chien *et al*. 2020). Although the HSI-II of *P. syringae* pv. DC3000 is not known to contribute to virulence against plant cells, it enables the bacterium to outcompete other microbes, increasing its fitness and allowing it to persist in the plant microbiota, thus increasing the chances that this bacterium will cause disease in the plant host. On the other side of this scenario, non-pathogenic bacteria with a T6SS have been proposed as possible biocontrol agents based on their ability to outcompete and inhibit plant pathogens. The environmental strain *Pseudomonas fluorescens* MFE01 was shown to outcompete *Pc atrosepticum* 6276 *in vitro* and *in planta*, protecting potato tubers from soft rot in a T6SS-dependent manner (Decoin *et al*. 2014). Similarly, *Pseudomonas putida* KT2440, which possesses three T6SS gene clusters, was shown to outcompete several plant pathogens *in vitro* in a T6SS-dependent manner, including *Xanthomonas campestris, A. tumefaciens, Pc carotovorum* and *P. syringae*. Furthermore, *P. putida* KT2440 was able to outcompete *X. campestris in planta* using a leaf infection model, protecting *Nicotiana benthamiana* from necrosis in a T6SS-dependent manner (Bernal *et al*. 2017). The impact of the T6SS on the microbiota can be more comprehensively investigated by studies that combine metagenomics analysis with *in vivo* competition approaches. For example, the plant pathogen *Burkholderia glumae* BGR1 was recently reported to have four T6SS gene clusters, with two of these clusters (T6SS group_4 and T6SS group_5) contributing to virulence towards rice plants when the bacterium is directly inoculated in the host, while one cluster (T6SS group_1) provides *B. glumae* BGR1 with antibacterial capacity. Metagenomic analysis of the endophytic bacterial community in rice plants inoculated with wild-type *B. glumae* BGR or a T6SS group_1-deficient mutant showed significant differences, with the overall taxonomic diversity in plants infected with the wild-type strain being lower and dominated by *Burkholderia*. Importantly, the abundance of specific bacterial genera such as *Luteibacter* and *Dyella* was specially decreased in a T6SS-dependent manner, suggesting specialized targeting by the secretion system (Kim *et al*. 2020). This last example highlights the importance of the T6SS in the plant ecosystem: it can be used by bacteria to shape and dominate the microbiota, and to increase virulence against the host.

The overall picture that emerges from these studies is that bacteria in the plant microbiota can likely use the T6SS against microbial competitors, the host plant, or both, depending on the T6SS effectors that a bacterium possesses. Although no T6SS effectors have yet been identified that are able to directly affect plant cells, assays that bypass microbial competition by inoculating pathogenic bacteria into plant tissues suggest that this secretion system may be involved directly in virulence against the plant host.

### The role of the T6SS in the gut microbiota

In recent years, enabled by faster and cheaper DNA sequencing technologies, the impact that the gut microbiota of an organism can have upon its health has become a subject of great interest (Otani, Chihade and Coopersmith 2019; Gomaa 2020). Various studies have shown that the members of the gut microbiota display distinct and unique behaviours when they are members of a community, and therefore studying them in isolation cannot fully reveal their relationship with their host. Indeed, the total metabolic activity of the gut microbiota is so diverse and rich that it has been called a ‘virtual organ’, comparable with the metabolic capacity of the liver in the case of human gut microbiotas (Shanahan 2002; O'Hara and Shanahan 2006). The gut environment represents a stable niche for microorganisms, protected from the harsh environmental conditions that may prevail in the exterior environment and with a relatively constant supply of nutrients provided by the diet of the host organism. Bacteria thus engage in fierce competition to occupy this desirable niche, and in this densely populated environment with up to 10^12^ bacteria/gram (O'Hara and Shanahan 2006), the T6SS can be used as an effective weapon to overcome competitors.

The human gut microbiota is dominated by anaerobic bacteria of the phyla Bacteroidetes and Firmicutes, which vastly outnumber bacteria of other phyla such as Proteobacteria, Verrucomicrobia, Actinobacteria, Fusobacteria and Cyanobacteria (Eckburg *et al*. 2005). T6SS gene clusters are common among Bacteroidetes, and those in the Bacteroidales have been classified into three distinct groups (GA1, GA2, and GA3) based on their genetic architecture (Coyne, Roelofs and Comstock 2016). An interesting characteristic of the GA1 and GA2 T6SS gene clusters is that they are widely distributed among Bacteroidales species of the gut microbiota, while the GA3 cluster is only found in *Bacteroides fragilis*. This has been attributed to the fact that the GA1 and GA2 gene clusters are contained within mobile genetic elements, which can be transferred among Bacteroidales species living in the gut environment (Coyne et al. 2014; Coyne, Roelofs and Comstock 2016). Of the three T6SS gene clusters common among Bacteroidales, only GA3 has so far been shown to convey antibacterial activity to the producing cells. Two strains of *B. fragilis* have demonstrated GA3-dependent killing ability against other Bacteroidales species, using *in vitro* and germ-free murine models (Chatzidaki-Livanis *et al*. 2016; Wexler *et al*. 2016). Interestingly, susceptibility to the GA3 system was limited to other Bacteroidales species, and the GA3 T6SS showed no antagonistic capacity against *E. coli* (Chatzidaki-Livanis *et al*. 2016). The effect of this selective killing pressure can be seen in the genetic makeup of the wider Bacteroidetes gut community, as some Bacteroidales species have acquired genes encoding immunity proteins to GA3 effectors even when they do not possess a GA3 T6SS cluster, thus gaining resistance against the attacks of *B. fragilis* (Wexler *et al*. 2016). Such “orphan” genes can be found encoded in large arrays known as acquired interbacterial defence (AID) gene clusters, which are contained in mobile genetic elements that can be transferred between Bacteroidales and confer resistance to T6SS activity (Ross *et al*. 2019). The overall impact of the GA3 T6SS of *B. fragilis* on the human gut microbiota has been studied in a metagenomic analysis, which revealed that in adult microbiotas, the *B. fragilis* population is dominated by a single strain with a small number of T6SS effector-immunity proteins. Furthermore, the study found that infant microbiota are more likely to contain strains of *B. fragilis* with a GA3 T6SS than adult microbiotas, which suggests that the infant microbiome is a highly competitive environment, with a single strain of *B. fragilis* emerging as dominant in the more stable adult microbiota and possibly then losing its GA3 gene cluster (Verster *et al*. 2017). The role of the GA1 and GA2 T6SS gene clusters remains to be defined, but their prevalence among Bacteroidales in the human gut microbiota and the observation that individual microbiomes contain only one, defined set of effector-immunity pairs, suggests that their main function may be to exert selective pressure for compatibility between multiple *Bacteroides* species and that they are maintained in the adult microbiome through HGT (Coyne *et al*. 2014; Verster *et al*. 2017). The overall conclusion of these examples is that, in the case of Bacteroidales, the T6SS seems to stabilize the microbiota by enforcing strain compatibility among members of this phylum.

T6SSs are found in several important human enteric pathogens such as *V. cholerae* (Pukatzki *et al*. 2006)*, Campylobacter jejuni* (Bleumink-Pluym *et al*. 2013; Kanwal *et al*. 2019)*, Shigella sonnei* (Anderson *et al*. 2017), and *S. enterica* (Sana *et al*. 2016), some of which have been shown to utilize their T6SS to outcompete or modify the resident microbiota, allowing them to colonize the host and cause disease. It was recently shown that 12 hours after inoculation of wild-type *V. cholerae* C6706, the intestinal load of *E. coli* in neonatal mice was up to 300-fold lower than that of mice inoculated with a T6SS mutant of C6706. Furthermore, mice inoculated with the wild-type *V. cholerae* strain showed an increase in biomarkers associated with infection such as interleukin 6 and the CXCL1 chemokine, while this did not happen in mice inoculated with the T6SS mutant; the same T6SS dependence was observed for disease symptoms such as diarrhoea (Zhao *et al*. 2018). Contrasting results with regard to the impact of the microbiota on T6SS function were observed with *V. cholerae* C6706 using a *Drosophila melanogaster* infection model. In that case it was shown that lethal cholera-like infection by *V. cholerae* C6706 was also dependent on its T6SS, however, the observed pathogenicity was dependent on the presence of the commensal bacterium *Acetobacter pasteurianus*, even though no T6SS-dependent killing of the commensal was observed *in vivo*. Removal of this commensal from the fly host abrogated T6SS-dependent killing by *V. cholerae* C6706, and reintroduction of the commensal restored the lethality of the *V. cholerae* C6706 infection. These results indicate that an interaction between *V. cholerae* and *A. pasteurianus* is necessary to cause a T6SS-dependent lethal infection in the fly host (Fast *et al*. 2018). This case suggests that signalling among members of the microbiota, or cellular contents from initially lysed microbiota cells, might serve as a cue to express T6SS activity.

*Shigella sonnei* has also been shown to utilize its T6SS to outcompete members of the commensal microbiota of mice, such as *E. coli*, *in vitro*. Furthermore, *S. sonnei* was dependent on its T6SS to persist in the gut environment, with the T6SS mutant being significantly cleared from the host in a 24-hour period. The antibacterial activity of the T6SS was shown to be responsible for this effect by pre-treating the mice with streptomycin to clear the local microbiota, in which case the T6SS mutant was able to colonise the host similarly to the wild type (Anderson *et al*. 2017). Similarly, *S. enterica* sv. Typhimurium has been shown to kill members of the mouse gut microbiota such as *Klebsiella oxytoca* and *Klebsiella variicola in vitro* via its T6SS SPI-6 gene cluster. This effect was augmented with the addition of bile salts, suggesting that the observed results may hold true in the gut environment. A functional SPI-6 T6SS was also shown to be necessary for *S. enterica* to persist in the host gut environment, with the T6SS mutant showing a 10-fold reduction in cell recovery from the host two days after infection as compared to the wild-type strain (Sana *et al*. 2016).

An indirect mechanism of microbiota manipulation by the T6SS was recently described in a patient-derived El Tor biotype of *V. cholerae* C6706. Using a zebra-fish model, this pathogen was shown to displace the commensal bacterium *Aeromonas veronii in vivo* in a T6SS-dependent manner. However, unlike other cases shown previously, the elimination of the commensal was not due to direct antibacterial activity of the secretion system. Instead, the T6SS was found to provoke alterations in the normal intestinal movements of the host which resulted in expulsion of the *Aeromonas* population. This function of the T6SS was linked to VgrG-1, a specialised effector with an actin cross-linking domain, expected to act on cells of the host intestinal lumen (Logan *et al*. 2018). The physical structure and organization of the intestines is also important for T6SS-mediated bacterial interactions because it presents different niches along its entire length, some of which have been shown to promote the cell-cell contact that is necessary for the delivery of most T6SS effectors (Fu, Ho and Mekalanos 2018). Overall, it is now clear that many enteric pathogens depend on their T6SS for successful colonisation of the host via interactions with the microbiota, generally, but not exclusively, via anti-bacterial activity against commensal species.

Beyond the human gut microbiota, the T6SS has been shown to influence microbiotas in insect hosts, with potential wider impacts for other higher eukaryotes in the same environment. *Pseudomonas protegens* CHA0, a member of plant microbiotas, depends on its T6SS to successfully invade the intestinal tract and haemolymph of the plant pest insect *Pieris brassicae*, causing a lethal infection. This invasion is mediated by the antibacterial activity of the T6SS, which enables *P. protegens* CHA0 to outcompete members of the host microbiota, mainly members of the *Enterobacteriaceae* family, which show a decline in their population during invasion by *P. protegens* (Vacheron *et al*. 2019). Thus, the T6SS may enable bacteria to function as biocontrol agents in particular plant-insect relations. T6SSs have also been shown to play important roles in the evolution of the gut microbiota of honey bees and bumble bees. The Beta-proteobacterium *Snodgrassella alvi* and the Gamma-proteobacterium *Gilliamella apicola* are important members of the microbiota of these insects and both of them possess T6SS gene clusters and associated effector-immunity gene pairs (Kwong *et al*. 2014). A family of T6SS specialised effectors known as Rhs proteins, large polymorphic toxins with N-terminal PAAR domains and variable C-terminal anti-bacterial toxin domains, was recently found to be highly prevalent and diverse among both *S. alvi* and *G. apicola*. A total of 1,112 *rhs* genes encoding 364 potentially distinct toxin domains were detected in just 77 analysed genomes of these two species (Steele *et al*. 2017). This diversity among Rhs effectors (and their immunity proteins) appears to result from HGT and homologous recombination. The *rhs* genes are commonly in proximity to integrase-like genes, which may serve to facilitate transfer of these effectors among strains of *S. alvi* and *G. apicola* (Steele *et al*. 2017). The diversity and abundance of Rhs effectors among *S. alvi* and *G. apicola* strains suggests a long co-evolution in the shared bee gut environment, with competitive dynamics and frequency-dependent selection preventing effectors from being lost. The effect of this conserved library of T6SS effectors on other bacterial species remains to be elucidated.

### The impact of the T6SS on marine niches

The marine environment shows a wide range of bacterial cell densities, from 10^3^ cells per ml in deep ocean waters to 10^6^ cells per ml in surface waters (Hobbie, Daley and Jasper 1977). As in other ecological environments, marine bacteria settle and colonize those niches that offer a better supply of nutrients and protection from changing environmental conditions. As seen in other environments, marine bacteria can utilize the T6SS as a useful tool for niche colonization and for shaping the microbiota within that niche.

*Euprymna scolopes* is a species of squid that possesses a specialized structure known as the light organ, which can be colonised by the bioluminescent bacterium *Vibrio fischeri* after the juvenile squid hatch, establishing a symbiotic relationship with their host (Ruby 1999). Although the diversity of *V. fischeri* strains in seawater is high and actually enriched by the squids, the light organ in an individual adult squid is inhabited by only a very small number of *V. fischeri* strains (Lee and Ruby 1994). It was recently shown that whilst *V. fischeri* strains all possess one T6SS gene cluster (T6SS1), only certain strains possess a second (T6SS2), which can be used to compete with other strains during light organ colonization *in vivo*. The light organ is divided into physically separated crypts, and each one can initially be colonized by only a few cells, which can be of different genotypes. When a crypt is co-colonized by strains of *V. fischeri* with and without the T6SS2, the cells of the T6SS2-lacking strain are eliminated from the crypt within 44 hours of the colonization (Speare *et al*. 2018). An interesting difference between *V. fischeri* and other commensals with a T6SS mentioned above is that the T6SS2 gene cluster shows a relatively low conservation among *V. fischeri* strains. This may reflect the fact that light organ crypts can still be successfully colonized by T6SS2-lacking *V. fischeri* strains if no other strain with a T6SS2 co-colonizes the same crypt (Speare *et al*. 2018; Guckes *et al*. 2019). Once the crypt is colonized, or when the cells are free-living in the ocean, the T6SS2 no longer provides a strong fitness advantage and instead the cells may face a fitness pressure to lose this large gene cluster.

A further example of T6SS-mediated microbiota manipulation and host colonization in the marine environment is found in *Vibrio vulnificus*. This bacterium can be ingested by *Crassostrea gigas* oysters during their normal seawater filtering if the bacterial cells are present in the organic particles that are consumed by the oysters. *Salmonella enterica* sv. Enteritidis is a also common member of oyster microbiotas (Tamber *et al*. 2020). Using a newly-developed *in vivo* model of oyster colonisation, *V. vulnificus* 106-2A was shown to cause a 10-fold decrease in the population of *S*. Enteritidis 24 hours after ingestion of organic particles carrying both organisms, with the deleterious effect on the *S*. Enteritidis population being dependent on the T6SS of *V. vulnificus* (Hubert and Michell 2020). The effects that the colonization of *V. vulnificus* may have on the host are unknown, however this bacterium is a well-known human pathogen that can cause serious infections from contaminated oysters (Phillips and Satchell 2017). Other *Vibrio* species have also been shown to be oyster pathogens and to depend on their T6SS to counter the immune response of the host. A study analysing various *Vibrio* strains commonly associated with *C. gigas* oysters found that the virulent *Vibrio tasmaniensis* strain LGP32 led to oyster death 24 hours after infection and caused hemocyte lysis *in vitro*, with both phenomena being drastically reduced in a T6SS1-deficient mutant (Rubio *et al*. 2019). Although the effect of the T6SS on the microbiota was not investigated, it was noted that *V. tasmaniensis* LGP32 possesses a second T6SS gene cluster (T6SS2) that was not involved in host virulence, but whose expression occurred within host tissues, suggesting that this second T6SS may be important during interactions with members of the host microbiota. *Vibrio parahaemolyticus* is another common inhabitant of marine environments that can be found in seawater or associated with various surfaces and hosts (Broberg, Calder and Orth 2011). This bacterium possesses two T6SS gene clusters. T6SS1 has been shown to provide antibacterial activity against *V. cholerae* and other Gamma-proteobacteria under simulated marine conditions at warm temperatures (Salomon *et al*. 2013). T6SS2, on the other hand, is expressed under low salinity conditions and has been associated with host-bacterium interactions, where it mediates the adhesion of *V. parahaemolyticus* to HeLa cells and induction of autophagy in macrophages (Yu et al. 2012, 2015; Salomon *et al*. 2013). Although the host-related functions of the T6SS2 have not been directly studied in marine hosts, the results obtained from these studies suggest that *V. parahaemolyticus* is an efficient bacterial competitor that uses its T6SS1 to eliminate members of the microbiota, which may in turn facilitate T6SS2-assisted colonization of the host.

Many marine bacteria are capable of adhering to various surfaces and forming biofilms, which offer them protection from various threats and allow them to display specialized behaviour (Dang and Lovell 2016). Once associated with these surfaces, bacteria can find themselves competing for resources with other bacterial species and strains. A recent study analysed various marine bacteria isolated from corals, oysters, sediment and seawater, challenging them with the pathogenic *V. cholerae* strain 2740–80 under simulated marine conditions. *V. cholerae* was shown to be an efficient bacterial competitor, depending on its T6SS to kill the competing cells. Gamma-proteobacteria were particularly susceptible, with decreases of up to 90% in their populations, whilst members of the Alteromonadales, Oceanospirillales, and Pseudomonadales were affected to a lesser extent (Guillemette *et al*. 2020). Interestingly, of all the tested strains, only *Vibrio coralliilyticus* was able to resist the T6SS of *V. cholerae* and was even able to kill this attacker. *V. coralliilyticus* also possesses a T6SS gene cluster, and a T6SS-deficient mutant strain of *V. coralliilyticus* was no longer able to kill *V. cholerae* nor survive its T6SS-mediated antibacterial activity. This suggests that *V. coralliilyticus* does not have an alternative resistance mechanism against the *V. cholerae* T6SS, but rather that it can simply kill *V. cholerae* faster or more efficiently using its own T6SS before *V. cholerae* has the chance to attack it successfully (Guillemette *et al*. 2020). In a similar fashion, other *Vibrio* species such as *Vibrio alginolyticus* and *Vibrio anguillarum*, have been independently shown to utilize their T6SSs in order to efficiently eliminate Gamma-proteobacteria under simulated marine conditions (Salomon *et al*. 2015; Tang *et al*. 2016).

As in other environments, the impact that the T6SS has upon these fierce inter-bacterial competitions is substantial and likely relevant to human disease. Many *Vibrio* species are pathogenic to shellfish or coral (Sussman *et al*. 2008; Cantrell *et al*. 2020), and they can have a major impact on human health through contamination of seafoods (Elbashir *et al*. 2018). It has been shown that several *Vibrios* utilize their T6SS to dominate local microbiotas and displace some of its members. It remains to be seen if, conversely, the T6SS can be used by niche commensals to prevent invasion of harmful *Vibrio* strains.

Beyond its role as simply an antibacterial weapon, in recent years a growing body of evidence has demonstrated that the T6SS plays further functions among bacterial communities in the marine environment that shape their composition and evolution. *V. cholerae* is found ubiquitously in seawaters, where it commonly colonizes and forms biofilms on chitinous surfaces such as the exoskeletons of zooplankton, which the bacterium utilizes as a nutrient source (Dang and Lovell 2016). Once growing on chitin, *V. cholerae* expresses gene clusters related to genetic competence and the T6SS, which form part of the same regulon controlled by the transcriptional regulators TfoX and QstR. This coordinated development of competence and production of a T6SS allows *V. cholerae* to kill neighbouring non-immune bacterial cells, releasing their DNA and enabling DNA uptake and natural transformation of *V. cholerae* itself. These natural transformation events occur at a much lower rate in T6SS-deficient strains (Borgeaud *et al*. 2015). The abundance of *V. cholerae* strains in seawater, together with their co-expression of competence and T6SS antibacterial activity, facilitates the transfer of genetic material among them. This creates a selection pressure for strains that can coexist in the same niche, which has led to the rise of compatibility groups. These groups comprise strains that share the same T6SS-associated immunity genes and can thus interact safely with one another (Kirchberger *et al*. 2017). Although compatibility groups are defined by shared T6SS effector-immunity gene pairs, orphan immunity genes are also found in *V. cholerae* that could enable a strain to survive contact with strains of a different compatibility group (Kirchberger *et al*. 2017). The high levels of transformation and recombination among *V. cholerae* strains in the marine environment have led to high genetic diversity in this species (Pretzer *et al*. 2017), with an emerging model of constantly shifting T6SS-defined compatibility groups that drive the evolution of the species and its adaptation to environmental challenges by the rapid spread of advantageous genetic traits among the population. In addition to this T6SS-facilitated genetic transformation, a family of *V. alginolyticus* T6SS effectors known as the MIX V clan has been shown to be associated with mobile genetic elements such as plasmids and transposons, leading to the hypothesis that these effectors may be horizontally shared among *Vibrios*. Indeed, a *V. parahaemolyticus* strain harbouring a plasmid encoding a *V. alginolyticus* MIX V effector was able to use this effector in T6SS-mediated competitions (Salomon *et al*. 2015). Although this effector-sharing seems to be limited by T6SS machinery compatibility (Salomon *et al*. 2015), it supports the perspective that *Vibrio* strains and species commonly engage in horizontal transfer of T6SS effector-immunity genes, which in turn influences the entire microbiota in the niches that they inhabit.

## GENERAL INTERACTIONS AMONG BACTERIA

Extensive work using laboratory models and domesticated strains has provided great insights into the structure of the T6SS, its effectors, and the impact the system can have on microbial interactions. Although these investigations normally do not employ conditions that approximate a natural environment, their conclusions allow careful speculation about how microbes may interact in their niches and can inform future research strategies more relevant to ‘real-life’ communities.

### Impact of the T6SS on the composition and evolution of bacterial genomes

As described above, T6SS activity has been shown to enhance HGT in *V. cholerae*. This phenomenon may occur widely in naturally competent bacteria. *Acinetobacter baumannii* is an opportunistic pathogen that has been designated a serious threat to public health due to its ability to rapidly acquire antibiotic resistance via HGT (Infectious Diseases Society of America 2004; ECDC/EMEA 2009). Similar to the case of *V. cholerae*, it has been shown that bacteria of the *Acinetobacter* genus utilize their single T6SS to facilitate the acquisition of new genetic traits. Using *Acinetobacter baylyi*, a close relative of *A. baumannii*, as research model, Cooper, Tsimring and Hasty (2017) demonstrated that this bacterium can efficiently lyse competing *E. coli* cells in a T6SS-dependent manner and acquire plasmids with fluorescent or antibiotic resistance markers harboured by the *E. coli*. This T6SS-enabled HGT was dependent on efficient lysis of *E. coli*, which in turn was influenced by the cell ratios during the competition: settings where the overall cell density was high and *A. baylyi* cells outnumbered the *E. coli* cells significantly enhanced HGT. Furthermore, it has been shown that this phenomenon is not simply dependent on the presence of a functional T6SS in *A. baylyi*; the delivered effectors must lead to cell lysis and the release of cellular contents. If the delivered T6SS effectors cause a decrease in the competitor's population without causing efficient cell lysis, the frequency of *A. baylyi* transformants is significantly lower (Ringel, Hu and Basler 2017). Interestingly, when T6SS-based competition between *A. baylyi* and *E. coli* cells is maintained for long time periods, the uptake of genomic DNA from lysed *E. coli* cells leads to the development of a subpopulation of filamentous *A. baylyi* cells that can reach >100 µm in length (normal *A. baylyi* cells are 1–2 µm long). This arrest of *A. baylyi* cell division was shown to depend on expression of competence genes and uptake of the genomic DNA released by T6SS-mediated cell lysis (Lin *et al*. 2019). In this case, the uptake of abundant non-homologous genomic DNA and concomitant activation of the SOS response was hypothesised to be the cause of cell division arrest, as the formation of filamentous cells happened at the interface region between *E. coli* and *A. baylyi* cells where the concentration of released genomic DNA is expected to be high (Lin *et al*. 2019). These findings indicate that finely-balanced regulation of competence development is necessary for *Acinetobacter* species to efficiently acquire new genetic traits by homologous recombination without sacrificing fitness.

Although the T6SS can facilitate the acquisition of genetic elements from competing bacteria by natural transformation, it can also be a hurdle for the safe dissemination of conjugative plasmids. *A. baumannii* strains commonly carry large conjugative plasmids (LCP) of up to 200 kbp that encode the conjugative machinery and multiple antibiotic resistance genes (Weber *et al*. 2015; Nigro and Hall 2017). Conjugation requires cell-cell contact for successful transfer of a plasmid to the recipient cell, with the survival of the recipient cell clearly also essential for propagation of the plasmid (Virolle *et al*. 2020). Contact-dependent T6SS antibacterial activity, expressed by the plasmid donor or recipient cell, can be counterproductive for conjugation. It was recently shown that LCPs carry genetic regulators that can inhibit T6SS expression in various *Acinetobacter* strains and species (Weber *et al*. 2015; Venanzio *et al*. 2019). This inhibition allows effective conjugation and dissemination of both LCPs and other small conjugative plasmids that do not possess the T6SS repressor (Venanzio *et al*. 2019). The emerging model is that *Acinetobacter* species can use their active T6SS to kill competing bacteria and quickly acquire new genetic traits either as plasmids or by homologous recombination, while LCPs can be safely disseminated via conjugation by inhibiting the T6SS. Although this model still needs to be further explored in more natural environments, the flexible DNA acquisition methods enabled by careful regulation of the T6SS suggests that *Acinetobacter* species are able to quickly adapt to environmental challenges and thus outcompete other members of the microbiota.

Finally, de Moraes *et al*. (2021) have recently reported an alternative mechanism by which the T6SS can modify bacterial genomes. DddA, a cytosine deaminase effector delivered by the T6SS of *B. cenocepacia*, can induce single-base-pair C→T mutations in *E. coli* cells following even a brief period of co-culture with *B. cenocepacia*. These mutations were shown to be ecologically relevant as they could provide *E. coli* with resistance to the antibiotic Rifampicin. This study highlights another important mechanism by which the T6SS may facilitate bacterial evolution and adaptation, particularly given the existence of a number of other, distinct families of candidate deaminase effectors (de Moraes *et al*. 2021).

### Quorum-sensing policing and intraspecies social control by the T6SS

Given the importance of the T6SS during microbial interactions and that its efficiency is linked to cell density (Cooper, Tsimring and Hasty 2017; Smith *et al*. 2020a), it is perhaps not surprising that this secretion system has been shown to be closely related to quorum sensing (QS) in several bacterial species. QS is a signalling system that allows bacteria to synchronize complex communal behaviour based on population density (Waters and Bassler 2005). In *P. aeruginosa* PA14 it has been shown that the QS-associated transcriptional regulators LasR and MvfR promote expression of the H2- and H3-T6SS gene clusters, while they inhibit expression of the H1-T6SS (Lesic *et al*. 2009; Maura *et al*. 2016). Similarly, in strain PAO1, expression of the H2-T6SS is upregulated as cell density increases, leading to augmented anti-host activity (Sana *et al*. 2012). The fact that the H1-T6SS has been shown to provide strong antibacterial activity (Hood *et al*. 2010) while the H2- and H3-T6SS have been associated with anti-host activity (Lesic *et al*. 2009) suggest a logic behind this differential regulation: During initial niche colonization it is advantageous for *P. aeruginosa* to express its H1-T6SS to compete with other members of the microbiota that may be present in higher numbers; whilst upon successful niche colonization, repression of H1-T6SS may be desirable since most *P. aeruginosa* cells will be surrounded by sibling cells resistant to its activity, and thus expression of this secretion system would represent a waste of resources. Conversely, expression of the H2- and H3-T6SS may be advantageous at that point in order to successfully invade host cells and tissues. The connection between QS and the T6SS has also been established in *Vibrio* species. Expression of the T6SS core gene *hcp1* in *V. alginolyticus* was shown to be under the control of QS regulators LuxO and LuxR, and to depend on the growth phase of the bacterium (Sheng *et al*. 2012). Similarly, expression of *hcp* has been shown to be growth phase-dependent and controlled by the QS regulators HapR and LuxO in the clinical *V. cholerae* strains A1552 and C6706 (Ishikawa *et al*. 2009; Zheng *et al*. 2010). Furthermore, under biofilm-forming conditions, the QS systems of *V. cholerae* A1552 promote expression of the competence regulon, which contains the T6SS genes (Borgeaud *et al*. 2015). Interestingly, this induction of competence and T6SS can happen in response to QS signals produced by various *Vibrios* within a multi-species biofilm (Antonova and Hammer 2011), further supporting the model of *V. cholerae* as a highly transformable species that benefits from interactions with other bacteria to drive its environmental adaptation. Further examples of the control of the T6SS by QS systems have been found in *B. thailandensis* (Majerczyk, Schneider and Greenberg 2016) and in *Burkholderia cenocepacia*, where QS also controls biofilm formation (Aubert *et al*. 2013). Interestingly, in the case of *B. thailandensis* it was shown that the BtaR1 QS system controls expression of T6SS effector and immunity genes that are found in loci outside the T6SS gene cluster, but it does not control expression of the T6SS core genes themselves. Within a population of *B. thailandensis*, a mutant strain that does not respond to BtaR1 QS signals would enjoy a fitness advantage by not participating in the costly metabolic functions associated with this QS system. Such ‘cheater’ cells could threaten to outgrow the QS cooperative cells and disrupt social behaviour. However, since the QS-defective mutant lacks expression of the immunity proteins and thus becomes susceptible to incoming effectors, the wild-type strain is able to kill the QS mutant in a T6SS-dependent manner, preventing the proliferation of cells that do not respond to QS signals (Majerczyk, Schneider and Greenberg 2016). This example highlights an important role that T6SSs can play within a bacterium's own population. Since QS systems regularly control the expression of common goods, the rise of cheater cells that do not produce the common good and grow faster is a risk to the entire population (Zhang, Claessen and Rozen 2016). Establishing QS-dependent expression of T6SS immunity proteins thus becomes an effective ‘policing’ mechanism to ensure that all the cells in the population participate in the shared social behaviour.

Another example of the T6SS being used as a mechanism of intra-species social control is found in *Proteus mirabilis*. This bacterium forms swarming colonies that form clear macroscopic ‘borders’ between the swarms of distinct strains, known as Dienes lines (Dienes 1946, 1947). It has now been shown that this phenomenon is T6SS-dependent (Alteri *et al*. 2013), and that minor variations in a single effector-immunity gene pair are sufficient to establish two otherwise isogenic strains as incompatible, enabling T6SS-mediated killing between them (Alteri *et al*. 2017). Thus, in *P. mirabilis* the T6SS can be used as a strong mechanism of kin discrimination, which may be necessary to guarantee the careful social coordination required during the swarming behaviour of this bacterium (Crespi 2001). A further case of T6SS-based kin discrimination has been reported in *Myxococcus xanthus*. This soil bacterium is capable of complex social behaviour, such as gliding motility and communal spore formation in fruiting bodies (Zhang *et al*. 2012). Troselj *et al*. (2018) showed that *M. xanthus* can utilize its T6SS to promote physiological synchronization of its population. In this case, physiological differences among cells caused by starvation lead to differential T6SS activity, with starving cells having reduced levels of immunity proteins. This allows T6SS-active cells to kill the starving sibling cells within the population. This mechanism may be beneficial for the *M. xanthus* population by allowing the cannibalization of less fit cells in order to delay or support the spore-formation process, which requires a major commitment of resources and important changes in gene expression (Zhang *et al*. 2012). This effect of the T6SS could have an impact on the evolution of this bacterium: cells able to cannibalize their siblings may also have additional mutations that increase their fitness, allowing them to maintain higher levels of T6SS immunity proteins, and would thus pass those mutations to their progeny.

### T6SS-mediated acquisition of nutrients

In recent studies it has been shown that certain T6SS-associated proteins do not function as toxic effectors but instead can be secreted into the extracellular milieu, where they can facilitate the sequestering and uptake of metal ions by the secreting cell. Under iron-deficient growth conditions, *P. aeruginosa* secretes the TseF effector to the extracellular medium. Secretion of TseF is largely dependent on the H3-T6SS of this bacterium, and once secreted it can interact with an iron-binding quinolone signalling compound produced by *Pseudomonas* and be incorporated into outer membrane vesicles. TseF then facilitates the capture of the sequestered iron by mediating interactions with membrane-bound receptors (Lin *et al*. 2017). In *B. thailandensis*, the T6SS-4 has been shown to secrete an effector, TseM, that functions as a manganese chelator and interacts with an outer membrane transporter to allow the import of manganese into *B. thailandensis* cells. This contact-independent function of its T6SS-4 helps *B. thailandensis* to overcome manganese starvation conditions and combat oxidative stress (Si *et al*. 2017b). Similar examples have been reported in other bacterial species where T6SS-secreted proteins participate in the sequestration and acquisition of copper (Han *et al*. 2019) and zinc (Wang *et al*. 2015; Si *et al*. 2017a). Although these studies have not examined the specific effects that metal ion sequestration have on the microbiota, competition for nutrients is well-known to be an important driver of microbial interactions (Ghoul and Mitri 2016; Bauer *et al*. 2018). In environments where bioavailable metal ions are scarce, efficient acquisition of those resources becomes a priority, and cells that successfully do so enjoy a fitness advantage that may allow them to outcompete other cells. Indeed Si *et al*. (2017a) showed that *B. thailandensis* utilises a T6SS-secreted zinc-chelating effector to gain a fitness advantage against Gram-negative and Gram-positive bacteria under conditions of zinc starvation. The ability of T6SS-secreted effectors to aid cells in this manner highlights the versatility of this secretion system as a highly advantageous trait for cells during interactions in the microbiota.

### Biofilm formation and the T6SS

Bacteria in various environments commonly form biofilms, where they display complex social behaviour, enjoy protection from harsh environmental conditions, and reach high population densities (Flemming *et al*. 2016). Due to the close proximity of cells living in a biofilm, T6SS-mediated interactions between cells could be expected to be important for this bacterial lifestyle. One of the main characteristics of biofilms is the production of exopolysaccharides (EPS) and other substances that envelop its cells, forming the so-called biofilm matrix. It has been shown that EPS produced by *V. cholerae* V52 cells can protect them from T6SS attacks from other cells, including siblings (Toska, Ho and Mekalanos 2018). This protection was due to the capacity of the EPS layer to block penetration by incoming T6SS attacks, rather than by neutralizing the effect of T6SS effectors. The protection provided by EPS is potent enough to allow significant survival of *V. cholerae* V52 cells lacking T6SS immunity proteins if its synthesis is highly induced (Hersch *et al*. 2020). A similar protective effect of EPS against T6SS attacks has been observed in *E. coli*. Expression of an EPS biosynthetic operon was induced in *E. coli* BW25113 cells when under attack by a T6SS-active *V. cholerae* strain and production of EPS enabled *E. coli* to survive better in this situation (Hersch *et al*. 2020). Conversely, various studies have reported co-regulation of biofilm formation and T6SS expression in *V. cholerae* (Borgeaud *et al*. 2015; Hersch *et al*. 2020), *B. cenocepacia* (Aubert *et al*. 2013) and other species (Sana *et al*. 2012). Biofilm formation is a complex endeavour that requires careful social coordination and different metabolic activities from cells living in different areas of the biofilm at different times (Flemming *et al*. 2016). Thus, it is possible that T6SS-based interactions may be necessary for biofilm initiation or its maintenance at different stages. Additionally, as-yet-unknown or contact-independent T6SS functions may be important for the cells living in a biofilm, such as cell signalling and sequestration of nutrients.

## INTERACTIONS WITH MICROBIAL EUKARYOTES MEDIATED BY THE T6SS

In addition to bacterial cells, microbial eukaryotes are also important components of many polymicrobial communities. Fungi are important degraders of organic matter in multiple environments, possess an outstanding diversity of unique metabolic pathways, and play an important role in maintaining the homeostasis of many organisms, including humans (Wisecaver, Slot and Rokas 2014; Seed 2015). Amoeba are protists commonly found in the microbiotas of water and soil environments, where they play an important role in shaping the microbial community by acting as bacterial predators, but can also establish symbiotic relationships with bacteria (Bonkowski 2004; Shi *et al*. 2020). It is now clear that bacteria can utilize the T6SS to mediate interaction with both of these eukaryotic neighbours.

The first T6SS-mediated interaction between amoeba and bacteria was reported in the study that originally coined the term ‘Type VI secretion system’. Pukatzki *et al*. (2006) showed that *V. cholerae* V52 depends on an active T6SS to resist *Dictyostelium discoideum* predation. Furthermore, this predation resistance is due to T6SS-mediated secretion of toxic effectors that kill the amoebal predator (Miyata *et al*. 2011; Dong *et al*. 2013). A more recent study revealed that the T6SS-dependent ability to survive *D. discoideum* predation and kill the amoeba is observed in various environmental clades of *V. cholerae*. This anti-amoebal T6SS activity is dependent on an actin-crosslinking effector but independent from anti-bacterial T6SS activity, which relies on distinct effectors (Drebes Dörr and Blokesch 2020). The plant pathogen *Xanthomonas citri* has also been shown to resist *D. discoideum* predation in a T6SS-dependent manner. Interestingly, in this bacterium, the T6SS does not appear to have antibacterial capacity, and expression of the T6SS gene cluster is promoted when *X. citri* is coincubated with the amoeba (Bayer-Santos *et al*. 2018). Although the signals that mediate the heightened T6SS expression are unknown, this bacterial response to the presence of a predator suggests a mechanism finely tuned by evolution that allows *X. citri* to survive and persist in hostile natural environments. Further cases of T6SS-mediated resistance to amoebal predation have been reported in the plant pathogen *P. syringae* (Haapalainen *et al*. 2012), the opportunistic pathogen *B. cenocepacia* (Aubert, Flannagan and Valvano 2008), and the intracellular pathogen *S. enterica* sv. Typhimurium (Riquelme *et al*. 2016). The case of *S*. Typhimurium is particularly interesting because the T6SS of this bacterium appears to assist with its survival and replication inside *D. discoideum* cells. This indicates that some bacterial species have adapted to not only avoid amoebal predation, but in fact use them as viable niches for proliferation. Indeed, *V. cholerae* has been reported to survive phagosomal lysis by *Acanthamoeba castellanii* and proliferate inside its vacuole, later promoting lysis of the amoeba to return to a free lifestyle (Van der Henst *et al*. 2016). In light of the importance of amoebal-bacteria relations for microbiota composition (Shi *et al*. 2020), the T6SS is revealed as a vital factor for shaping the microbiota by enabling specific bacterial species to thrive among amoebal predators, while other are consumed as prey.

Despite the fact that fungi share ecological niches with bacteria in multiple environments (Seed 2015; Bonfante, Venice and Lanfranco 2019), T6SS-based interactions between fungi and bacteria have been poorly studied. A transcriptomics study of *P. fluorescens* provided the first indication of a possible T6SS-based interaction by showing that the expression of its T6SS gene cluster increased upon exposure to roots infected by the pathogenic fungi *Gaeumannomyces graminis* (Barret *et al*. 2009). The first case of direct T6SS-mediated antifungal activity was reported in the plant pathogen *P. syringae*, which can suppress the growth of the yeast *Cryptococcus carnescens* in a T6SS-dependent manner (Haapalainen *et al*. 2012). More recently, the first specialized antifungal effectors were described in the opportunistic pathogen *Serratia marcescens*, which produces two distinct T6SS-secreted effectors that can kill or disrupt the metabolism of the yeasts *Candida albicans*, *Candida glabrata*, and *Saccharomyces cerevisiae* (Trunk *et al*. 2018). Although an initial examination of bacterial genomes indicated that T6SS antifungal effectors are conserved in several bacterial species (Trunk, Coulthurst and Quinn 2019) and T6SS antifungal activity has recently also been suggested in *Klebsiella pneumoniae* (Storey *et al*. 2020), further research is needed to clearly establish if T6SS antifungal capacity is widespread among bacteria and, perhaps more interestingly, if non-antagonistic T6SS-mediated interactions may exist among bacteria and fungi.

## CONCLUSION AND PERSPECTIVES

Although the history of the T6SS research field is relatively short, we have seen a remarkable expansion in our understanding of its mechanism and, more importantly, in our appreciation of its versatility of function and breadth of influence. It is now clear that the T6SS represents a vital tool for interbacterial competition, microbiota control, host manipulation, and contact-independent competitive fitness in very many, diverse Gram-negative bacterial species. Use of the T6SS can shape microbial communities, determine the outcome of host-bacterial interactions, both directly and indirectly, and even drive bacterial evolution. This leads to the prediction that the T6SS will have a substantial impact on the ecological dynamics of diverse bacteria in many realms of nature. To address this prediction and further expand our understanding of this bacterial system, many important questions and exciting areas remain to be explored. These include:

- The impact of the T6SS has, to date, been mostly studied using limited competition approaches between only two bacterial strains. Further modelling and experimental investigations are needed to fully understand the impact of the T6SS on complex microbial communities and consortia.
- T6SS-based interactions have only been explored among a small number of microorganisms and ecological niches. The impact of this system on many further environments, such as alkaline springs (with their archaeal inhabitants) and woodland soils (with their exuberant abundance of fungi and bacterial species) needs to be explored.
- It has been shown that the T6SS can serve as an intra-species mechanism for social control under specific circumstances. It remains to be elucidated if this usage is more widespread and, specifically, if the T6SS can be used to deliver signalling molecules to sibling cells or can serve to coordinate complex social behaviour such as biofilm formation.
- How the T6SS is used in conjunction with other bacterial competition mechanisms remains an open question. It will be interesting to study the evolutionary dynamics that cells may undergo when growing under conditions that allow multiple competition strategies.
- A detailed understanding of the molecular mechanisms and regulation of the T6SS should ultimately allow for its use in biotechnological and biomedical applications. From biocontrol and targeted anti-pathogen therapeutic organisms (Ting *et al*. 2020) to molecule delivery and independent exploitation of T6SS effectors, the possibilities will continue to expand as we learn more about this versatile system.

We anticipate that many fascinating discoveries lie ahead for this research field, that will continue to develop our perspective of the T6SS and its multifaceted role in microbial ecology.
